# Developing Oncolytic Viruses for the Treatment of Cervical Cancer

**DOI:** 10.3390/cells12141838

**Published:** 2023-07-13

**Authors:** Eleni Kalafati, Ekati Drakopoulou, Nicholas P. Anagnou, Kalliopi I. Pappa

**Affiliations:** 1 Laboratory of Cell and Gene Therapy, Centre of Basic Research, Biomedical Research Foundation of the Academy of Athens (BRFAA), 11527 Athens, Greece; 2 First Department of Obstetrics and Gynecology, University of Athens School of Medicine, 11528 Athens, Greece

**Keywords:** oncolytic viruses, cervical cancer, virotherapy, viral vectors, cancer immunotherapy, innate immunity, adaptive immunity

## Abstract

Cervical cancer represents one of the most important malignancies among women worldwide. Current therapeutic approaches for cervical cancer are reported not only to be inadequate for metastatic cervical cancer, but are also considered as cytotoxic for several patients leading to serious side effects, which can have negative implications on the quality of life of women. Therefore, there is an urgent need for the development of innovative and effective treatment options. Oncolytic viruses can eventually become effective biological agents, since they preferentially infect and kill cancer cells, while leaving the normal tissue unaffected. Moreover, they are also able to leverage the host immune system response to limit tumor growth. This review aims to systematically describe and discuss the different types of oncolytic viruses generated for targeting cervical cancer cells, as well as the outcome of the combination of virotherapy with conventional therapies. Although many preclinical studies have evaluated the therapeutic efficacy of oncolytic viruses in cervical cancer, the number of clinical trials so far is limited, while their oncolytic properties are currently being tested in clinical trials for the treatment of other malignancies.

## 1. Introduction

Cervical cancer (CC) represents one of the major causes of death among women worldwide, with about 530,000 new cases diagnosed and 275,000 deaths per year [1,2]. The most crucial etiological factor is the infection from high-risk human papilloma virus strains (hrHPV), followed by other determinants, such as age, smoking, childbirth, use of oral contraception, and diet [3]. In the last decade, CC incidence rates and deaths in the developed countries have gradually declined, as a result of cancer screening tests and the vaccination strategies against hrHPV [4,5]. Indeed, the most effective approach for the prevention of CC includes vaccination to prevent HPV infections during adolescence, followed by screening to detect HPV infections during adulthood [6]. However, it is worth noting that, due to the lack of effective prevention and/or screening methods, the incidence of CC is still increasing in developing countries [7].

A persistent hrHPV infection is not sufficient to immortalize and transform the cervical epithelial cells of the host; the existence of genetic and epigenetic alterations has been shown to be required for the development of carcinogenesis [8]. Thirteen percent of CC patients are diagnosed at advanced stages, while the 5-year survival rate for metastatic CC is 16.5% compared to 91.5% for the localized disease [9]. Patients who are diagnosed with early-stage CC or locally advanced CC have access to conventional treatments that comprise surgery, chemotherapy or radiotherapy [10]. However, the treatment options for patients with metastatic CC are quite limited because of its heterogeneous manifestations. Current targeted therapies, such as angiogenesis and immune checkpoint inhibitors, were not able to significantly increase the overall survival [11,12,13,14].

**Figure 1:**
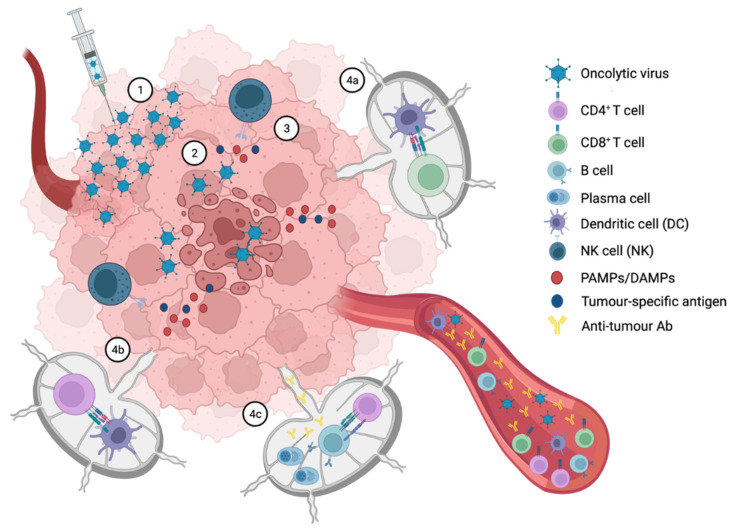
Primary events following OVs’ administration, triggering both oncolysis and anti-tumor immunity **1**. OVs are administered either intratumorally or intravenously **2**. Following transduction, OVs kill tumor cells by lysis, inducing the release of viral progeny, tumor-specific antigens (TSAs), pathogen-associated molecular patterns (PAMPs) and damage-associated molecular patterns (DAMPs) **3**. Innate immunity and immunologic cell death are mediated primarily by NK cells and dendritic cells (DCs) **4**. Antigen presentation by DCs takes place in the draining lymph nodes, where tumor-specific antigens are presented to CD8 **^+^** T cells (**4a**), CD4**^+^** T cells (**4b**) and B cells (**4c**). Then, CD4**^+^** and CD8**^+^** T cells become activated and start to proliferate, while B cells upon activation, differentiate into plasma cells, and start producing tumor-specific antibodies.

Oncolytic virotherapy is a promising alternative therapeutic strategy for incurable cancers. Oncolytic viruses (OVs) are genetically engineered or naturally occurring viruses that selectively replicate intracellularly and eventually kill cancer cells, without harming normal tissues. As explained in Figure 1, their multiple mechanisms of action include not only direct cell lysis, but also the stimulation of the host antitumor immunity.

Several oncolytic viral products have been approved so far. Specifically, IMLYGIC™ (talimogene laherparepvec), a second-generation oncolytic herpes simplex virus type 1 (HSV-1), is the first oncolytic viral therapy approved by the US Food and Drug Administration for the treatment of metastatic melanoma in 2015 and subsequently in Europe in 2016 [15]. Other OVs generated from different parental viruses have also been tested in Phase III clinical trials with outstanding results, such as Pexa-Vec (pexastimogene devacirepvec), an oncolytic vaccinia virus (VV), CG0070 an oncolytic adenovirus (AdV), and REOLYSIN™ (pelareorep), an oncolytic reovirus [16]. For the purpose of CC oncolytic virotherapy, the main viruses used are adenoviruses, herpes viruses, parvoviruses and the Newcastle disease virus. In this review, we systematically discuss the development of OVs as an innovative therapeutic approach for the treatment of CC, as well as their therapeutic efficacy and safety, as demonstrated by preclinical and clinical trials. The OVs utilized as therapeutic agents for CC are summarized in Table 1.

## 2. Adenoviruses

**Figure 2:**
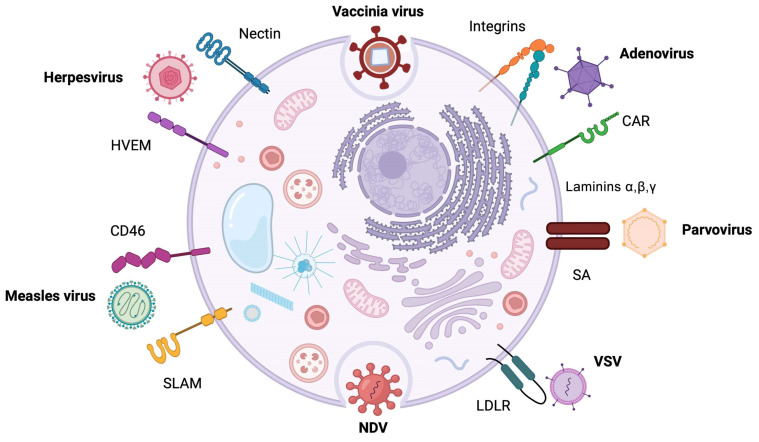
Oncolytic viruses recognize and invade the target cells through the interaction with specific host cell surface receptors. CAR, Coxsackie virus and adenovirus receptor; SA, sialic acid moieties; VSV, vesicular stomatitis virus; LDLR, low density lipoprotein receptor; NDV, Newcastle disease virus; SLAM, signaling lymphocytic activation molecule; HVEM, herpesvirus entry mediator.

They represent the most frequently used viruses for oncolytic virotherapy in cancer, including cervical cancer. AdVs are DNA viruses common in animals and humans, with a linear, non-integrating, double-stranded DNA (dsDNA) genome ranging from 30 to 38 kb [17]. The size of their icosahedral capsid ranges from 70 to 100 nm, with more than 100 serologically different types identified [18]. The first attempt to use an oncolytic AdV was made by Bauerschmitz et al. [19], who utilized a replication-competent AdV, designated as Ad5-Delta 24 RGD. The Ad5-Delta 24 RGD virus can preferentially replicate in defective cells in the Rb-p16 molecular pathway, which is the case for most cancer cells [19]. This is a case of conditionally replicating AdVs (CRADs), which benefit from such tumor-specific modifications, allowing preferential replication in tumor cells [20]. Other approaches take advantage of the recognition of tumor-specific receptors for transducing cancer cells (Figure 2). However, the expression level of the Coxsackie-adenovirus receptor (CAR) fluctuates and most epidermal-derived normal tissues express CAR, thus, the use of an untargeted AdV would lead to the transduction of mainly non-target cells [20].

Ad5-Delta 24 RGD carries a 24-bp deletion in the constant region 2 (CR2) of the *E1A* gene, which makes the protein product unable to bind the retinoblastoma (Rb) tumor suppressor/cell cycle regulator protein [19]. This binding allows AdV to induce S-phase entry, and thus, viruses with this type of modification lose their ability to overcome the G1-S checkpoint and replicate aptly in cells where this molecular mechanism is disturbed [21]. The fiber of Ad5-Delta-RGD was also modified by incorporating an αvβ3 and αvβ5 integrin-binding arginine-glycine-aspartic acid (RGD-4C) motif into the HI loop of the knob domain, in order to increase AdV tropism to tumor cells [19]. Moreover, RGD-modified AdVs partially evade preexisting humoral immunity [22]. Interestingly, the expression of these integrins is retained in cervical carcinogenesis, with frequent overexpression seen in advanced tumor progression [23]. Ad5-Delta 24 RGD generated a significant therapeutic effect not only in vitro in C33A, CaSki, HeLa, and SiHa cervical cancer cell lines, but also in vivo in a C33A murine model employing 10^8^–10^10^ virus particles, with no detectable cytotoxicity in peripheral blood mononuclear cells of the infected mice [19].

Another genetically engineered oncolytic AdV is the AdCB016-mp53 virus, which was designed to selectively replicate in HPV-containing cells. AdCB016-mp53 carries the *p53* gene variant *mp53* (268N), which is resistant to HPV E6-mediated degradation, while AdCB016 replicates selectively in cells expressing HPV E6 and E7 proteins, due to two deletions in its E1A protein, which prevent viral replication in normal cells. E1A protein is responsible for reprogramming the infected cell to promote virus replication [24,25]. In an organotypic raft culture, AdCB016-mp53 killed SiHa cells, while leaving normal epithelium unaffected [24]. Kim et al. [26] generated eleven E1A mutant AdVs with deletion or substitution of the E1A Rb-binding sites. One of these mutants, Ad-E1mt7, in which both the *E1A* and *E1B* genes were deleted, demonstrated significant efficiency in cytotoxicity and viral replication in a tumor cell-specific manner. Moreover, the antitumor efficacy of Ad-E1mt7 was demonstrated in a C33A xenograft model, where tumor growth was inhibited. The survival rate dramatically increased, while it is worth noting that two of the nine mice treated with Ad-E1Bmt7, showed a complete response without signs of regrowth, even three months following their treatment [26].

Squamous cell carcinoma antigen-2 (SCCA2) expression is related to a poor prognosis [27]. Hsu et al. [27] used a luciferase reporter assay to prove that the SCCA2 promoter was active in human CC cell lines, such as Cx, Cxwj, SiHa, and HeLa cells, but relatively quiescent in normal cervical epithelial cells. Next, they developed a CRAD AdV vector, named Ad-KFH, which carries the E1B 55 kDa deletion and in which the viral *E1A* gene was under the transcriptional control of the SCCA2 promoter. The replication of Ad-KFH was restricted to cervical cancer cells, while Ad-KFH treatment of mice bearing peritoneal Cxwj-derived tumors resulted in tumor growth retardation and prolongation of their survival. Of note, the combination of Ad-KFH with cisplatin, dramatically increased the survival of tumor-bearing mice, exhibiting a synergistic effect between the two remedies [28]. Another research group investigated the therapeutic efficacy of AdVs harboring the cyclooxygenase-2 (*Cox-2*) or the vascular endothelial growth factor (VEGF) gene promoter to control replication. Their hypothesis was based on the fact that anti-inflammatory agents can lower Cox-2 protein levels and thus control AdVs’ oncolytic activity. It was demonstrated that both promoters could be downregulated either with dexamethasone, sodium salicylate, or salicylic acid. Although the inhibitory effect of dexamethasone was not strong enough to provide significant differences in an in vivo environment, a good therapeutic efficacy of the viruses was documented following intravenous administration in a metastatic CC murine model [29].

Xiao et al. [30] constructed a selectively replicating AdV with the *E1B* gene deletion (ZD55), designated ZD55-VEGI-251, in which a secreted isoform of vascular endothelial cell growth inhibitor (VEGI-251) was inserted. VEGI is a member of the tumor necrosis factor family and acts as an inhibitor of endothelial cell proliferation and angiogenesis [30]. They tested the oncolytic capacity of ZD55-VEGI-251 in cervical, hepatoma, and colorectal cancer cell lines. Indeed, ZD55-VEGI-251 reduced cancer cell viability in an autocrine-dependent manner as a result of mitochondria-mediated apoptosis, accompanied by caspase-9 and caspase-3 activation and PARP cleavage. Remarkably, no caspase-8 enhancement was observed. In a model of a HeLa human cervical tumor, ZD55-VEGI-251 led to 80% suppression of tumor growth and inhibited angiogenesis. Furthermore, assessment of the activity of crucial liver enzymes, such as aspartate aminotransferase (AST), alanine aminotransferase (ALT), and alkaline phosphatase (ALP) in the sera of the experimental animals, documented no generalized cytotoxicity [30].

Ad.sp-E1A(D24)-IL-24 is an oncolytic AdV which is not only characterized by a 24-bp deletion in the *E1A* gene, but also by the inclusion of the interleukin 24 *(IL-24*) gene. The recombinant virus replication was driven by the promoter of the *BIRC5* gene which encodes survivin [31]. This approach exploited IL-24 antitumor capacity and the promoter of survivin, which is overexpressed in almost all human tumors, but is rarely detectable in normal cells [32,33]. Ad.sp-E1A(D24)-IL-24 increased cytotoxicity only in tumor cells (PLC, HeLa, NCI-H1299, and NCI-H460) at low MOIs of 0.1, 1, 5, and 10. Additionally, the cytopathic effect of Ad.sp-E1A(D24)-IL-24 was about 100 times greater than that of Ad-IL-24 in the NCI-H1299 and NCI-H460 cell lines. Infection of NCI-H460 cells with Ad.sp-E1A(D24)-IL-24 and Ad.spE1A(D24), led to extensive morphological changes related to apoptosis, such as chromatin condensation, nuclear fragmentation and generation of apoptotic bodies. In contrast, and regarding normal WI38 and L-02 cells, significant cytotoxicity was observed only at the highest MOI of 100 [31].

A different approach by Wang et al. [34] involved the design of an E1A-mutant AdV (M6) with antisense HPV 16 E6/E7 DNA inserted into the deleted 6.7K/gp19K region of the AdV E3 gene. Both *E6* and *E7* genes encode oncoproteins that act by interfering with tumor suppressor proteins, such as p53 and pRB. M6 was able to inhibit the expression of *E6* and *E7* oncogenes, induce apoptosis and reduce the invasion ability of HPV16-positive SiHa cells. On the contrary, M6 could not inhibit the expression of the HPV *E6* and *E7* oncogenes in the HPV16-negative CC cells HeLa and C33A, while the induction of apoptosis was much lower compared to SiHa cells. In vivo experiments demonstrated that M6 transfection remarkably improved the survival of tumor-bearing mice in combination with radiotherapy [34]. A similar strategy was followed by the same research team in the construction of the M5 oncolytic AdV which employs HPV E2, an apoptosis-inducing agent and a crucial negative transcriptional modulator of HPV *E6* and *E7* oncogenes, under the control of the *E3* gene promoter [34,35]. M5 also carries a 27-bp deletion in the E1A CR-2 region, in order to achieve tumor-specific replication. M5 preferentially silenced the HPV *E6* and *E7* oncogenes in HPV-positive CC cells and exhibited antitumoral efficacy both in vitro and in vivo, while the therapeutic effect was augmented in combination with radiation. Of interest, M5 also increased cytotoxicity in HPV-negative cells, mainly due to the pro-apoptotic ability of the E2 protein [35].

Wang et al. [36] modified the p53-targeted AdV to become radiation-responsive. In their approach, the oncolytic AdV was constructed by inserting a radiation-responsive expression cassette composed of the promoter of the early growth response-1 (*Egr-1*) gene and the gene for the pro-apoptotic protein TNF-related apoptosis-inducing ligand (*TRAIL*). This approach shared the same backbone with H101 AdV that carries the E1B55K-/E3 deletions. H101 was designed to target the *p53*-mutated cells and has been approved in China for the intratumoral treatment of several malignancies, including CC [36,37]. The combination of H101 at a very high MOIs of 1000 or 10,000, combined with radiotherapy, led to a synergistic anticancer effect in SiHa, CaSki, and HeLa cells with the maximum effect achieved in the HPV-negative C33A cells [37]. The Egr-1/TRAIL AdV reinforced cell death and induced apoptosis at MOI 100. Furthermore, in mice bearing xenograft tumors, intratumoral administration of Egr-1/TRAIL, accompanied by radiation, diminished tumor growth and enhanced mice survival [36].

A recent study utilized an oncolytic AdV named ZD55-TRAIL, which comprises the *TRAIL* gene, along with the histone deacetylase inhibitor suberoylanilide hydroxamic acid (SAHA) [38]. SAHA is an inhibitor of class I and II histone deacetylases (HDACs), which are responsible for the deacetylation of histones and other proteins, maintaining chromatin in a more relaxed state, and thereby allowing transcription of genes that are involved in carcinogenesis [39]. These agents synergistically kill HeLa cells by inducing G2 growth arrest and apoptosis. Specifically, the apoptotic rate of HeLa cells co-treated with ZD55-TRAIL and SAHA was roughly three times higher than that of ZD55-TRAIL treatment alone. Notably, ZD55-TRAIL induced the activation of the extrinsic apoptotic pathway enhancing the activation of caspase-8, caspase-3, and cleavage of PARP; the activation of this pathway was further enhanced by the co-treatment with SAHA [38]. It was also found that SAHA inhibits ZD55-TRAIL-induced upregulation of IκBα, p50, and p65, which are crucial molecules in the NF-κB pathway that regulates cellular growth and proliferation [38,39,40]. The ZD55-TRAIL therapeutic outcome was further confirmed in a cervical tumor xenograft model established by HeLa cells. Consistent with the in vitro results, ZD55-TRAIL plus SAHA demonstrated the highest growth suppression in parallel to increased apoptosis [38].

## 3. Herpes Viruses

Human herpes viruses (HSV) include human simplex type 1 and 2, Epstein–Barr virus, Kaposi’s sarcoma-associated herpesvirus, cytomegalovirus, etc. The Herpesviridae family is characterized by a spherical virion that comprises four major components: the core, the capsid, the tegument, and the envelope. The core contains a single copy of a linear, dsDNA molecule packaged at high density into the capsid. The diameter of the virion is approximately 200 nm and depends on the viral species [41]. HSV has multiple mechanisms to evade immune responses, thus, can be genetically modified and used as a powerful anti-tumor weapon to target tumor cells. Cellular entry of HSV involves binding to several transmembrane receptors [42], such as herpes entry mediator (HVEM) and nectin-1, as shown in Figure 2.

Several pre-clinical studies have demonstrated the efficacy of HSV in CC [43]. The HSV-1 virus hrR3, is defective for the large subunit of the ribonucleotide reductase (ICP6 or UL39); however, its replication is complemented by the capacity of tumor cells to preferentially upregulate ribonucleotide reductase, a feature missing in post-mitotic normal cells. The oncolytic virus hrR3 when combined with ionizing radiation results in complementary toxicity in malignant cell lines [44,45]. Similarly, recombinant strains of HSV-1 containing mutations in the infected cell protein (ICP) 34.5, have been shown to replicate preferentially in rapidly proliferating tumor cells, causing a direct cytolytic effect in a dose-dependent fashion. Moreover, subcutaneous C33A tumors in SCID mice were significantly reduced by 50%, following a HSV-1 mutant (G207) intratumoral treatment. Furthermore, combination therapy with a low dose of radiation resulted in 42% complete eradication of the tumor [44]. To further investigate the therapeutic efficacy of HSV-1, Kagabu et al. [46] evaluated the therapeutic effect of a triple-mutated oncolytic HSV (T-01) in HPV-related CC cell lines and immunodeficient or immunocompetent mouse models [46]. The triple-mutated HSV G47Δ was constructed by generating a further deletion in the *α47* gene and the overlapping the *US11* promoter of the G207 genome. Τhe *α47* gene encodes a protein responsible for inhibiting the transporter associated with antigen presentation (TAP), and its absence led to increased MHC class I expression in infected cells. G47Δ exhibited higher replication capability, a partial restoration of MHC class I expression and a greater antitumor effect compared to G207 [46,47]. Based on the above, T-01 was constructed with a similar structure to G47Δ. T-01 was highly cytotoxic in vitro, while in the HeLa xenograft and the TC-1 syngeneic models, it led to a significant reduction in tumor growth. Notably, increased numbers of CD8^+^ T-cell precursors in the T-01-treated mice group were observed, probably due to T-01 infection. Furthermore, T-01 demonstrated an immunoregulatory function, since MHC class I expression was increased. However, further studies are required to elucidate the mechanism underlining this process [46].

An oncolytic virus with significant oncolytic properties and a broad antitumor spectrum is bovine herpesvirus 1 (BoHV-1). Its therapeutic outcome was demonstrated in a human lung adenocarcinoma cell line and a xenograft mouse model wherein it suppressed tumor cell proliferation and growth [48]. According to our knowledge, BoHV1 has not been tested as an oncolytic virus for the treatment of CC. However, its tropism for cervical medulla [49] would make it a potential therapeutic virus in the aforementioned cancer type.

## 4. Newcastle Disease Virus

Non-human viruses are sought after, since they could retain their ability to induce lysis in specific host cells—traditionally not susceptible to them—and at the same time they are considered to be non-pathogenic [50]. Newcastle disease virus (NDV) represents such an example, since it can selectively replicate in tumor cells and exert direct cytotoxic effects on them [51], entering the cytoplasm of target cells by endocytosis (Figure 2). NDV is an avian, enveloped, negative-sense, ssRNA virus of the Paramyxoviridae family. The 15 kb non-segmented RNA genome comprises six genes that encode for six structural proteins, namely the nucleocapsid protein (NP), the phosphoprotein (P), the fusion protein (F), the hemagglutinin-neuraminidase surface glycoprotein (HN), the envelope matrix protein (M), and the large protein (L) [52]. Successful use of the NDV as a potent oncolytic agent, has been demonstrated in several studies, using strains, such as the MTH68/H, LaSota, Anhinga, PV701, and AF2240-I [53,54,55]. NDV has anti-neoplastic and immune stimulatory properties causing immunogenic cell death and systemic anti-tumor immunity. Of special interest is the fact that localized oncolytic NDV virotherapy has been shown to overcome systemic tumor resistance to immune checkpoint blockade immunotherapy [56]. Furthermore, clinical trials confirmed the infrequent side effects and a high safety profile, since the first report of using NDV as a treatment in a patient with acute leukemia was published in 1964 [57,58,59,60,61,62].

NDVs are classified into three pathotypes according to the chicken pathogenicity: lentogenic (avirulent), mesogenic (intermediate), and velogenic (virulent). LaSota and Hitchner B1 (HB1) are lentogenic strains that are exploited as live vaccines against Newcastle disease [63]. Keshavarz et al. used the NDV vaccine strain La Sota to infect TC-1 cells of an HPV-associated cervical cancer model of C57 mice which express HPV-16 E6/E7 antigens [64]. The TC-1 cell viability decreased almost 50% at MOI 20–40, and the maximum release of OV NDV was achieved 72 h after infection at MOI 40, coinciding with the maximum apoptosis induction. The apoptosis induced by NDV in TC-1 cells has been suggested to be mediated mostly by the intrinsic apoptosis pathway, while caspase-9 increase was noted [64].

The oncolytic properties of the other lentogenic stain HB1 were also assessed as a potent therapeutic agent against CC. HB1 NDV infection (MOIs 5, 10, and 15), led to production of reactive oxygen species (ROS), enhancing apoptosis and autophagy induction in TC-1 cells, in a dose-dependent manner [65]. Moreover, NDV significantly upregulated the expression of cytochrome C and downregulated the expression of survivin, a suppressor of apoptosis that belongs to the IAP protein family, associated with cell survival via inhibition of caspase activity [63,65]. The maximum release of HB1 OVs was also noted 72 h post-infection at a significantly low MOI 15, leading to increased apoptosis. Similarly, microtubule-associated protein 1 light chain 3 (LC3) was altered from LC3-I to LC3-II, highlighting the autophagy activation [65]. Nevertheless, the mechanism by which NDV caused ROS production and induced autophagy remains unknown.

It has been demonstrated that some viral fusogenic membrane glycoproteins (FMGs) could enhance viral propagation and increase the infection of tumor cells by OVs. Based on the hypothesis that the incorporation of influenza hemagglutinin-2 (HA2) FMG could improve the therapeutic outcome of NDV against CC, Miri et al. [66] generated a NDV vector harboring HA2 (NDV-HA2). The tumor size of the NDV-HA2-treated mice in weeks 4, 5, and 6 was significantly reduced compared to the NDV-treated group. Interestingly, mice treated with NDV-HA2 displayed a remarkable lymphocyte proliferation response, while cytokine secretion assay revealed an increase in IFN-γ and IL-12 [66]. This outcome indicates that NDV infection may induce Th1 cytokines that play a crucial role in amplifying the antitumor cellular immune response [66,67]. Additionally, the enhancement of IL-4 secretion underlined the induction of Th2 cell differentiation [66,68]. Meanwhile, the increased levels of granzyme B, a serine protease most commonly found in natural killer cells (NK cells) and cytotoxic T cells, also revealed the role of cellular immune responses. On the contrary, the secretion of IL-10 and the transforming growth factor β (TGF-β) was suppressed in the tumor microenvironment in NDV-HA2-treated mice, suggesting that NDV is capable of reducing T regulatory cell (Treg) activity [66], as well.

## 5. Parvoviruses

The Parvoviridae family includes 134 small ssDNA viruses with genomes of around 5 kb [67,68]. These viruses infect a variety of animals, ranging from invertebrates to mammals, causing disease in some hosts or subclinical infections in many others. The different subfamilies of Parvoviridae comprise the parvoviruses (Parvovirinae) and the densoviruses (Densovirinae) [69]. Among OVs, the parvoviruses deserve special consideration for their promising anticancer properties, particularly the rat oncolytic H-1 parvovirus (H-1PV) which has been the therapeutic agent in many studies in melanoma, breast cancer, pancreatic cancer as well as cervical cancer [70]. Recent data [71] have documented that laminin heterotrimeric complexes comprising α (1-5), β (1-4) and γ1 laminin are required for the H-1PV cell attachment and entry occurring via their sialic acid moieties (Figure 2). The outcome of the first attempt to exploit H-1PV as a potential therapy in cervical carcinoma was published in 2013. In that study, H-1PV was used with valproic acid (VPA), a histone deacetylase inhibitor [72]. VPA is a widely used drug that acts directly at the level of gene transcription by suspending the deacetylation of histones and rendering transcription sites more accessible, and therefore, altering the expression of many genes [73]. VPA synergizes with H-1PV to kill cervical cancer cells HeLa, CaSki, SiHa, and early passage tumor cell cultures, and by increasing the levels of ROS as well as DNA damage, leads to apoptosis. Remarkably, in HeLa xenograft mice treated with both agents, complete eradication of the established tumors was documented [72].

Interestingly, H-1PV is the first Parvoviridae family member to undergo clinical testing as an antitumor therapy [74]. Results from clinical trials in patients with recurrent glioblastoma and pancreatic cancer, confirmed that H-1PV virotherapy is safe and well-tolerated, while H-1PV treatment led to improved progression-free survival and median overall survival of glioblastoma patients [74,75]. Further investigation on the aspects of the H-1PV life cycle would not only advance the field of virology but also could help improve the H-1PV efficacy as OV. The entry route of H-1PV in HeLa cells has been studied employing electron and confocal microscopy. The H-1PV particles were detected within clathrin-coated pits and vesicles, suggesting that the virus entry is mediated through clathrin-mediated endocytosis [75].

To overcome the potential side effects of OVs and improve the therapeutic outcome, Saxena et al. constructed a bicistronic vector, named pVIVO.VP3.NS1, that carries the genes for VP3 protein (apoptin) of chicken infectious anemia (CIA) and NS1 (non-structural protein 1) protein of canine parvovirus-2 (CPV-2), which have shown oncolytic potential [76]. Apoptin is a proline-rich protein capable of activating apoptosis mostly in tumor cells. In non-malignant cells, apoptin accumulates towards the cell margins, but is eventually degraded by proteasome, without harming the cells. On the contrary, in cancer cells, a cancer cell-specific kinase phosphorylates apoptin accumulates in the nucleus and forms multimers, inhibiting the DNA repair mechanism, thereby forcing cancer cells to undergo apoptosis [77]. NS1 protein of CPV2 has also been proven to induce caspase-dependent and p53-independent apoptosis in cancer cells, while no toxic side effects on healthy cells have been reported [78,79]. The transduction with pVIVO.VP3.NS1 led to a significant increase in apoptosis (43.6%) in HeLa cells, suggesting a synergistic apoptotic effect of both NS1 and VP3 proteins [76].

## 6. Other Viruses

Other studies have utilized OVs, such as a genetically engineered vaccinia virus (VV), the canine distemper virus or the influenza B virus, and provided further evidence that these viruses can efficiently transduce CC cells and induce apoptosis [80,81,82]. Specifically, Goncharova et al. tested in vitro a genetically modified VV that carries the transgene of GFP protein, named LIVP-GFP, both in vitro in human cervical carcinoma and other cancer cell lines and in vivo in mice bearing tumors. Remarkably, the administration of LIVP-GFP not only inhibited tumor growth but also suppressed metastasis formation [80].

Sindbis virus (SINV) was also tested as a potential therapeutic agent for CC [83]. SINV is a generally non-pathogenic alphavirus. SINV contains a non-segmented, positive-sense ssRNA genome with a 5′ cap and a 3′ poly(A) tail, and has been used as a model to define alphaviruses life cycle determinants [84]. The infection of two CC cell lines with SINV led to a strong cytopathic effect and apoptosis, compared with reovirus infection at the same low MOI, while in normal human keratinocytes the cytopathic effect was very low for both viruses. Of interest, a low dose administration of SINV in both HeLaS3 and C33A mice tumors led to a significant reduction in tumor size compared with the mock-treated mice. Moreover, the overall survival of SINV-treated animals was dramatically increased [83].

The Edmonston strain of measles virus (Edm-MV) shows remarkable oncolytic activity against a variety of human tumors including CC, targeting tumor cells mainly through the CD46 receptor, as depicted in Figure 2. Edm-MV is capable of triggering apoptosis in infected tumor cells and inhibiting tumor growth in mice. Notably, caspase 3, a key mediator of apoptosis, can accelerate viral replication in CC cells and augment the cytopathic effects of Edm-MV. On the contrary, deficiency of caspase-3, either in tumor cells or in tumor xenograft models, considerably prevents oncolysis with Edm-MV [85]. It seems that apoptosis is a double-edged sword. Specifically, it has been documented that drug-induced apoptosis can improve the oncolytic effect of MV; however, apoptosis via activation of caspase-3 during radiation can stimulate tumor repopulation [86,87]. Undoubtedly, MV is considered an oncotropic virus with promising therapeutic properties. However, adopting this virus as an anti-cancer therapeutic tool raises important concerns due to the fact that preexisting antibodies in vaccinated patients against MV can neutralize it [88].

## 7. Clinical Trials

In 1956, the National Cancer Institute (NCI) conducted the first pioneer clinical study, administering wild-type AdVs to thirty women with CC achieving a varying degree of tumor necrosis, but no significant tumor regression. Although early studies like the aforementioned were considered groundbreaking, no further attention was given to the use of viruses as potential antineoplastic therapies, mainly due to the moderate efficacy and the unacceptable side effects that raised considerable concerns. Ultimately, the emergence of modern genetic engineering in the 1990s allowed the concept of viral oncolysis to resurface with renewed potential as an alternative cancer therapy [89].

To date, approximately thirty OVs are being explored either as monotherapy modality or in combination with other anticancer treatments, with more than 120 clinical trials published [90]. To our knowledge, no clinical trial has been completed that assesses the therapeutic efficacy of OVs specifically against CC (clinicaltrials.gov; accessed on 3 July 2023). The first phase I clinical trial for intratumoral injection of the recombinant oncolytic type II HSV, BS-006 in CC, was first posted in May 2022 and is going to be completed in July 2024 (NCT05393440). Recently, in April 2023, an early phase I clinical study was announced on oncolytic virus for the treatment of relapsed/refractory cervical and endometrial cancer (NCT05812677). This study utilizes intratumoral or intraperitoneal injection of an oncolytic recombinant HSV-1, R130. The R130 vector carries the gene coding for anti-CD3 scFv/CD86/PD1/HSV2-US11 in order to induce T-cell cytotoxicity [91]. The study is estimated to include 20 participants and its results are expected to be published in 2026. However, CC patients have participated in clinical trials that evaluated the efficacy and safety of OVs against solid tumors. A study that utilized intravenous injection of PV701, a Newcastle disease virus, has showed that the virus is well-tolerated and reported four major and two minor tumor responses. Eighteen patients were enrolled in this study, receiving a median of six PV701 cycles, ranging from 2 to 16 cycles. Of note, the only CC patient enrolled in the above study showed complete response for more than 30 months [92]. A minimal overall response was reported in a phase I clinical trial that tested the combination of intravenous administration of reovirus type 3 Dearing (RT3D) in patients with advanced solid tumors including two CC cases [93]. Reovirus antitumor efficacy was also tested in a two-stage phase I dose-escalation study of intratumoral injection of Reolysin^®^—also an oncolytic reovirus—combined with palliative radiation, and it exhibited partial responses in both low and high dose groups. Two of the sixteen patients enrolled in the study that evaluated Reolysin^®^ suffered from CC, but further information about their response was not available [94].

Ad5/3 delta 24 (Ad5/3-Δ24) is an oncolytic AdV being studied for putative applications across a number of neoplasms, and potentially with gynecologic malignancies. This OV targets cancer cells through binding to the Ad3 receptor, which is overexpressed in cancer cells, compared to the normal neighboring cells. Twenty-one patients with gynecologic malignancies received intraperitoneal injection of the OV. The treatment with Ad5/3-Δ24 was well tolerated with only grade 1–2 adverse events of fatigue, malaise, and abdominal pain, while 71% of patients achieved no disease progression for a couple of months [95]. Moreover, Ad5/3–Δ24 was tested in a phase I clinical trial against recurrent ovarian cancer, where it was demonstrated that almost 30% of women had a decrease in CA-125 (cancer antigen 125) levels at one month, while the adverse events, such as fever, myalgia, fatigue, and nausea were also moderate [96].

Another phase I clinical trial has explored the therapeutic efficacy of an engineered MV which expresses the carcinoembryonic antigen (MV-CEA) in recurrent ovarian cancer. In this study of 21 patients, more than half demonstrated stable disease, whilst five had a marked decrease in CA-125 levels, following intraperitoneal injection of MV-CEA. Interestingly, the median overall survival increased compared to historical controls [97].

A recent case report described the successful treatment of a 19-year-old patient suffering from clear cell adenocarcinoma of the uterine cervix, with recombinant human adenovirus type 5 (Oncorine^®^), formerly known as H101. The patient was first treated with external beam radiotherapy and chemotherapy with imperceptible reduction in the tumor. However, the combination of the intratumoral injection of Oncorine^®^ with brachytherapy and chemotherapy, led to complete response after a seven-month follow-up with no serious adverse events [98].

Lastly, there are many trials for OVs that exhibit promising results for treating head and neck cancer (HNC), which is another HPV-related cancer. Among them, adenovirus plays a key role. Similarly, the deletions of cytopathic viral genes allow for the selective replication in HPV-infected cells. A phase I clinical trial of a conditionally replicating AdV armed with the gene encoding GM-CSF showed that intratumoral administration was safe and well tolerated, while the number of patients was not sufficient to assess its effectiveness [99]. GM-CSF was also used in an oncolytic HSV-1 vector, in a phase I/II study in head and neck cancer patients, combined with radiotherapy and cisplatin, and it exhibited a locoregional control with a high relapse-free rate (76.5%) [100]. Reovirus, a naturally occurring non-pathogenic which has natural oncolytic activity, is being evaluated in phase I-III clinical studies in a variety of tumors. The combination of reovirus and chemotherapy shows impressive responses, while reovirus monotherapy exhibited partial responses and disease stabilization in relapsed/metastatic head and neck cancer [101]. Despite the differences in the pathology of HNC and CC, they appear to share common HPV-driven oncogenic pathways, and thus, it would be interesting to test the reovirus efficacy in CC cell lines and preclinical models.

## 8. Discussion

The promising preclinical studies obtained with OVs so far provide the impetus for further development of this approach, especially for the aggressive cancer types for which the current therapeutic options are grossly inadequate. Preclinical trials of engineered OVs in CC have been widely initiated and have shown promising results. Remarkably, Ad5-Delta 24 RGD, an oncolytic AdV, exhibited great therapeutic outcome in a mouse xenograft model with no detected side effects, while partially avoiding preexisting antibodies [19]. Similarly, ZD55-VEGI-251 AdV induced an 80% suppression of tumor size as well as the inhibition of tumor angiogenesis [30]. The main advantages of using AdVs are based on the fact that they are well-studied and that their natural diversity can be exploited by increasing OV tropism. On the contrary, attenuated viral spread requires a higher MOI to achieve therapeutic outcome, while antiviral immunity limits efficacy [102,103]. Importantly, other OVs, such as the HSV T-01, can cause complete eradication of the tumor in the majority of tumor-bearing mice, not only through their ability to rapidly replicate in cancer cells but also by activating the immune system [46]. The genetic modification of HSV allows it to replicate only in tumor cells. However, its larger capsid compared to other OVs may hinder its systemic administration with the potential suppression of OV-mediated antiviral immunity [103].

A fundamental prerequisite for the development of OVs is to limit their replication within malignant cells. Parvoviruses and NDVs are naturally occurring OVs which exert their selective tumor replication without the necessity of further genetic modifications. On the contrary, OVs, such as AdVs, require genetic manipulation of viral genes to enhance tumor selectivity and reduce virus pathogenicity [104]. This strategy is based on the deletion of viral genes that are necessary for efficient replication in normal cells but are dispensable in tumor cells. For instance, in AdVs, the deletion of the *E1B* 55K gene blocks the cell cycle regulator p53, and thus limits the virus replication to occur only in tumor cells where the function of *p53* gene has already been lost [105]. AdVs and other DNA viruses, such as HSV and PV, have the advantage of a larger and more stable genome, facilitating the genetic engineering and addition of multiple transgenes. However, DNA viruses exhibit a lower immunogenicity compared with some RNA viruses [106].

Another consideration in the development of OVs is their tropism for tumor cells through the recognition of specific receptors. MVs as a characteristic example, bind to the CD46 receptor which is expressed at high levels on many human cancer cells [107]. Although most OVs demonstrate acceptable safety and tolerability in clinical studies, infection of non-cancer cells by OVs raises concerns. Of note, cells of the tumor endothelium are susceptible to HSV infection. After early infection, HSV targets endothelial cells inhibiting angiogenesis. However, as a result of the rebound effect, the angiogenic pathway is upregulated mainly through VEGF induction. In the case of AdVs, high susceptibility of hepatocytes to AdV infection is associated with increased expression levels of the virus entry receptors. AdV, but also NDV and MV, demonstrate high viral titers in secondary lymphoid tissues, especially in spleen [108]. All of these above-mentioned factors could be a serious concern for developing anticancer therapies based on OVs. However, the infection of normal cells by OVs is tolerable and could be exploited to enhance the antitumor immunity.

Arming OVs with immunostimulatory cytokines has been a popular approach to generate immunological synergy coupled with the effect of oncolysis, can achieve high response rates. The addition of *IL-24* gene to Ad.spE1A(D24) increased the therapeutic outcome with no significant cytotoxicity as it was confirmed in CC cells. In clinical studies, the combination of OVs with immunomodulators, monoclonal antibodies or checkpoint inhibitors, seems to yield increased potency and long-term benefits in some cancer patients, underscoring the need for further improvement. The combination of OVs with traditional remedies, such as chemotherapy or radiation, has been investigated in preclinical trials in a rational way to improve the eventual treatment benefits for patients suffering from CC. Of interest, the combination of oncolytic AdVs with chemotherapy or radiotherapy, exhibited a synergistic effect between the two remedies by increasing the survival of tumor-bearing mice [28,34,85]. Moreover, in the case of HSV-1, the combination with radiation led to a complete eradication of tumors in almost half of the animals involved in the study. In addition, the combination of the oncolytic T-01 virus with the anti-programmed cell death ligand 1 (PD-L1) antibody, increased the number of tumor-specific T-cells in the tumor microenvironment, as it was demonstrated in a TC-1 murine model [109].

On the contrary, adoptive cell therapy as monotherapy has shown limited efficacy for solid tumors. OVs, on the other hand, have the unique ability to decrease the immunosuppression within the tumor microenvironment, facilitating immune cellular responses. Consistent with this observation, the tumor microenvironment becomes more favorable for adoptive cell therapy. Following OV infection, tumor cell lysis releases tumor-specific antigens transforming a previous immunologically *cold* tumor into a *hot* one that recruits a series of effector immune cells, as demonstrated in mouse models, leading to an upgraded immunity [110]. Based on the above findings, it is anticipated that future well-designed clinical trials employing these combinatorial approaches can convincingly confirm the therapeutic advantages of the combination of these promising novel strategies.

## 9. Conclusions

OVs are a promising therapeutic tool for the treatment of patients who present recurrent cervical cancer. Preclinical studies have proven their efficacy in mouse xenografts not only in combination with traditional treatment options, such as chemotherapy or radiation, but also as a monotherapy approach. The limited number of CC patients who have participated so far in clinical trials that tested OVs against solid tumors, had a positive therapeutic benefit with no severe toxicity. However, clinical practice is a more complex process, and more clinical studies should be conducted to provide evidence about the effectiveness and safety of OVs. More promising results are anticipated in the near future by combining OVs with existing cancer therapies, since OVs have the ability not only to kill tumor cells but also to upgrade and accelerate the anti-tumor immune responses. However, additional well-designed future studies will be necessary for the eventual application of OVs in personalized gynecological cancer therapy by also exploiting their additional ability to serve as gene therapy vectors [111].

**Table 1:** Engineered oncolytic viruses for cervical cancer.

**Virus**	**Name**	**Gene Modification**	**Outcome**	**Ref.**
**Adenovirus**	Ad5-Delta 24 RGD	▪ 24-bp deletion in the *E1A* gene	▪ increased tropism to tumor cells ▪ avoidance of preexisting humoral immunity ▪ significant therapeutic effect in C33A, CaSki, HeLa, and SiHa cells and in a C33A murine model with no detectable cytotoxicity	[19,22]
	AdCB016-mp53	▪ *mp53* variant resistant to HPV E6-mediated degradation ▪ two deletions in its *E1A* genes	▪ killing of SiHa cells while leaving normal epithelium unaffected in an organotypic raft culture	[24,25]
	Ad-E1mt7	▪ deletions in the *E1B* gene	▪ tumor growth inhibition in a C33A xenograft mouse model	[26]
	Ad-KFH	▪ the *E1A* gene under the transcriptional control ofthe *SCCA2* gene promoter	▪ growth retardation and prolongation of the survival in a Cxwj xenograft mouse model	[28]
	RGDCRADcox-2R	▪ the *E1A* gene under the transcriptional control ofthe *cox-2* gene promoter	▪ efficient killing of C33A, SiHa, HeLa and CaSki cells ▪ therapeutic efficacy in a C33A murine model ▪ virus replication partially controlled by dexamethasone, salicylic acid and sodium salicylate	[29]
	Ad5/3VEGF-E1	▪ the *E1A* gene under the transcriptional control of the *VEGF* promoter	▪ efficient killing of C33A, SiHa, HeLa and CaSki cells ▪ therapeutic efficacy in a C33A murine model ▪ virus replication partially controlled by dexamethasone, salicylic acid and sodium salicylate	[29]
	ZD55-VEGI-251	▪ E1B 55 kDa deletion and insertion of the *VEGI*-251 gene	▪ reduction in cancer cell viability as a result of mitochondria-mediated apoptosis ▪ 80% suppression of tumor growth and inhibition of angiogenesis in a HeLa xenograft model with no major cytotoxicity	[30]
	Ad.sp-E1A(D24)-IL-24	▪ 24-bp deletion in the *E1A* gene and insertion of the *IL-24* gene ▪ virus replication was driven by the *survivin* gene promoter	▪ increased cytotoxicity in HeLa cells	[31]
	AdV-M6	▪ antisense *E6 E7* DNA of HPV 16 inserted into the deleted 6.7K/gp19K region of the *E3* gene	▪ enhanced apoptosis and reduction in the invasion ability of HPV16-positive SiHa cells ▪ improved survival of tumor-bearing mice in combination with radiotherapy	[34]
	AdV- M5	▪ the HPV *E2* gene under the control of the *E3* gene promoter	▪ antitumoral efficacy both in vitro and in vivo, while the therapeutic effect was augmented in combination with radiotherapy	[34,35]
	Egr-1/TRAIL AdV	▪ backbone of an E1B55K-/E3-deleted H101 AdV ▪ insertion of the *TRAIL* gene under the control of the *Egr-1* promoter	▪ intratumoral administration accompanied by radiotherapy diminished tumor growth and enhanced survival in a HeLa-S3 mouse model	[36]
	H101	▪ E1B55K-/E3 deletions	▪ anticancer effect in SiHa, CaSki, HeLa, and C33A cells	[37]
	ZD55-TRAIL	▪ insertion of the *TRAIL* gene	▪ ZD55-TRAILin combination with SAHA demonstrated growth suppression and increased apoptosis in HeLa cells	[38]
**Herpes virus**	hrR3	▪ defective for the *rR* gene	▪ combined with ionizing radiation resulted in complementary toxicity in CaSki cells	[45]
	G207	▪ a deletion of the two copies of the *γ34.5* gene and an inactivating insertion of the *LacZ* gene replacing the *ICP6* gene	▪ 42% eradication of tumor in combination with low-dose radiation in mice	[44]
	T- 01	▪ a deletion of the *γ34.5* and *α47* genes and an inactivating insertion of the *LacZ* gene replacing the *ICP6* gene	▪ highly cytotoxic for HeLa and CaSki cells ▪ significant reduction in tumor growth in HeLa xenograft and TC-1 models	[46]
**Newcastle disease virus**	La Sota		▪ TC-1 cells viability decreased with parallel induction of apoptosis	[64]
	Hitchner B1		▪ ROS production, enhanced apoptosis and autophagy induction in TC-1 cells	[65]
	NDV-HA2	▪ incorporation of the influenza *hemagglutinin-2* gene	▪ tumor size decrease with lymphocyte response and secretion of IFN-γ and IL-12 ▪ promotion of Th2 cell differentiation	[66,68]
**Parvovirus**	H-1PV		▪ H-1PV synergized with VPA to kill HeLa, CaSki, SiHa and CxCa cells, increasing the levels of ROS and causing apoptosis ▪ complete eradication of the established tumors in a HeLa xenograft mouse model treated with both agents	[72]
**Lentiviral** **vector**	pVIVO.VP3.NS1	▪ incorporation of the *VP3* and *NS1* genes	▪ significant increase of apoptosis in HeLa cells	[76]
**Vaccinia** **virus**	LIVP-GFP	▪ insertion of the *GFP* gene	▪ inhibition of tumor growth and metastasis formation	[80]
**Sindbis virus**	SIN AR339		▪ low-dose administration leads to a significant reduction in tumor size in HeLaS3 and C33A mouse xenografts	[83]
**Measles virus**	Edm-MV		▪ inhibition of tumor growth and induction of apoptosis in SiHa mouse xenografts	[85]
